# Emergence of Wrinkles during the Curing of Coatings

**DOI:** 10.3390/gels4020041

**Published:** 2018-05-03

**Authors:** Michiko Shimokawa, Hikaru Yoshida, Takumi Komatsu, Rena Omachi, Kazue Kudo

**Affiliations:** 1 Fukuoka Institute of Technology, Fukuoka 811-0295, Japan; s13e2070@bene.fit.ac.jp (H.Y.); s13e2027@bene.fit.ac.jp (T.K.); 2 Department of Computer Science, Ochanomizu University, Tokyo 112-8610, Japan; omachi.rena@is.ocha.ac.jp (R.O.); kudo@is.ocha.ac.jp (K.K.)

**Keywords:** paint coating, wrinkle, swelling, buckling

## Abstract

Wrinkles often emerge on a paint layer when a second coat of paint is applied on an already-coated substrate. Wrinkle formation occurs when the first layer absorbs organic solvent from the second layer. We set up experiments to mimic the double-coating process, focusing on the interaction between a paint layer and an organic solvent. In the experiments, we investigated the characteristic wavelengths of the wrinkles and the time of wrinkle emergence. We employed a simple model to explain the wrinkle emergence and performed numerical simulations. The linear stability analysis of the model provides a relation between the wavelengths and the characteristic timescale that agrees reasonably well with our experimental data as well as numerical results. Our results indicate that compression of the layer due to swelling and delamination are both important factors in the formation of wrinkles.

## 1. Introduction

Double coating, in which paint is applied over an already-coated substrate, is often used to avoid unevenness in the paint layers. In spite of double coating, however, wrinkles sometimes emerge in the drying process, if (1) the elapsed time between the first and second coatings is too short, or (2) the coat thickness is too great [1,2]. The formation of wrinkles has been studied in many fields, such as engineering, material science, chemistry, and physics [3]. The fundamental process of wrinkle formation, however, is not yet fully understood. Below, we focus on case (1) above and investigate the formation of wrinkles from the viewpoint of the mechanical stability of the paint layer.

In a double-coating process, two layers of paint are produced by the first and second coatings. Deformation of the first layer, underlying the second layer, leads to the formation of wrinkles observed at the surface of the second layer. A resin paint, which includes a polymer and an organic solvent, is often used in the painting process. The mechanism of wrinkle formation by a resin paint is thought to be as follows:A polymerization reaction proceeds in the layer after the first application, and the stiffness of the layer increases as it cures [4]. When a second coating is applied, the organic solvent, which is an ingredient in the second coating of paint, penetrates into the first layer. Exposure to the organic solvent causes the polymerized first layer to swell [5]. The first layer is easily swollen when the elapsed time between the first and second coatings is too short, because of incomplete polymerization of the first layer [6]. The swelling induced by absorption of the solvent thus causes deformation of the first layer, and wrinkle formation at the surface of the second layer is due to the resulting deformation of the underlying first layer. Most previous experiments on double coating have focused on the top (second) layer rather than the first layer [1,7], and the effect by the deformation of only the first layer has not been investigated quantitatively.

In this paper, we propose an experiment in which an organic solvent is applied to the surface of the first layer to mimic the double-coating process. We can then observe the deformation of the first layer directly in the experiment, without the complicated effects of the deformation and polymerization of the second layer. The emergence of wrinkles in this experiment is due solely to the deformations caused by the instability of the first layer. We investigate the characteristic length scales, i.e., the wavelengths, of the wrinkles, together with the characteristic timescale that characterizes wrinkle formation. The characteristic lengths and the timescale depend on the elapsed time *T* between the application of the first coating and the application of the organic solvent. In order to investigate the emergence of wrinkles, we employ a simple model that includes both the effects of buckling due to the swelling of the layer and delamination of the layer from the substrate. The model provides a relation between the wavelengths and the characteristic timescale. The relation is demonstrated as novel types of plots of our experimental and numerical results. The result indicates that the swelling of the layer and its delamination from the substrate cause the instability of the layer that leads to the emergence of wrinkles. In the following sections, we discuss experimental and numerical data in detail and consider the process of wrinkle formation by means of a model.

## 2. Results and Discussion

### 2.1. Experimental Results

#### 2.1.1. Buckle Formation

**Figure 1:**
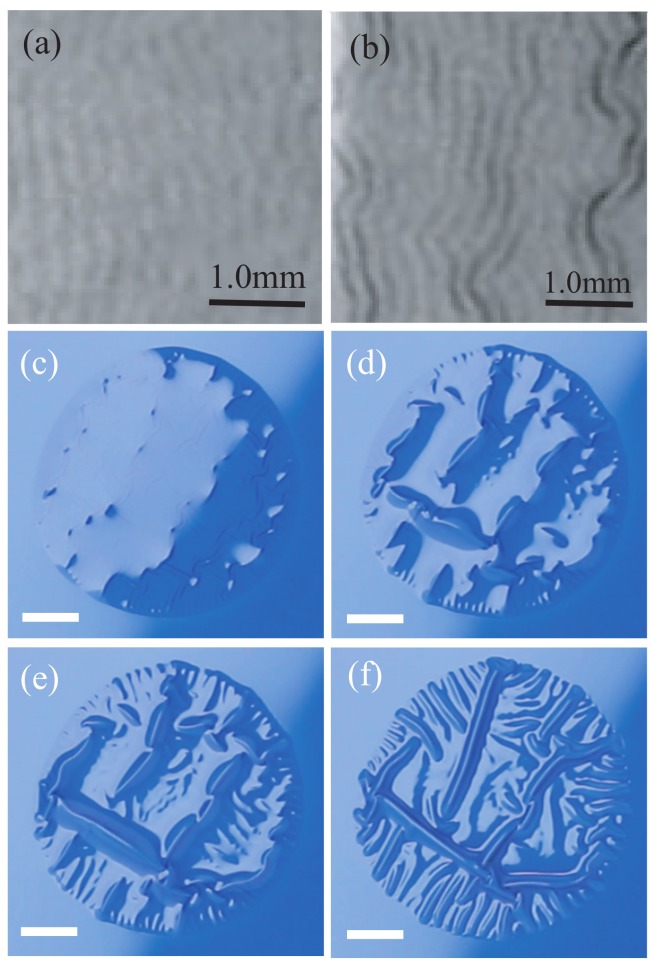
Deformations of the paint layer at (**a**) t=58 s, (**b**) 63 s, (**c**) 90 s, (**d**) 120 s, (**e**) 150 s, and (**f**) 300 s, where t=0 is the time when the organic solvent is applied to the layer. The contrast of (**a**,**b**) is modified only for clearer demonstration of wrinkles. These photographs all apply to experiments for which the curing time T=24 h. The solid lines in panels (**a**,**b**) are 1.0-mm-scale bars, and those in panels (**c**–**f**) are 3.0-mm-scale bars.

Figure 1 shows the deformations of the paint layer at the following times *t* after application of the organic solvent: (a) t= 58 s, (b) 63 s, (c) 90 s, (d) 120 s, (e) 150 s, and (f) 300 s, all for experiments at the fixed time T=24 h after the application of the paint layer. The drop of organic solvent spreads into a circular shape approximately 11 mm in diameter. Short-scale wrinkles first emerge at t=58 s (Figure 1a). Shortly after that, at t=63 s, larger-scale wrinkles appear (Figure 1b), and small bumps appear randomly at t=90 s (Figure 1c). The bump amplitudes are much larger than those of wrinkles observed at earlier times. The amplitude of the bumps increase with *t*, and delamination of the layer from the substrate occurs. In the process, some bumps coalesce with other bumps (Figure 1d,e). The coalescence repeats, and buckles emerge, as shown in Figure 1f. The pattern of buckles does not change after t=300 s.

**Figure 2:**
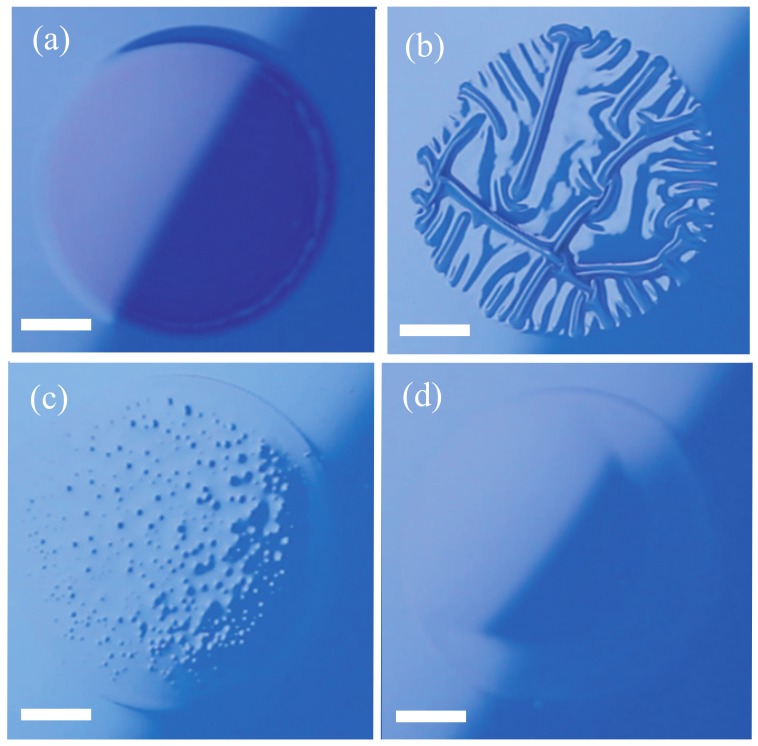
Deformations of layers obtained in experiments for (**a**) T=1 h, (**b**) 24 h, (**c**) 56 h, and (**d**) 64 h, where *T* is the time elapsed between the application of the paint layer and the application of the drop of organic solvent. These images were taken at t=600 s after the application of the organic solvent. Solid lines in the photos are 3.0-mm-scale bars.

We focus here on the deformations of the paint layer that occur in experiments for several different values of the curing time *T*. Figure 2 shows snapshots for (a) T=1 h, (b) 24 h, (c) 56 h, and (d) 64 h. These images were all obtained at t=10 min. Buckles emerge only in experiments for T=24 h (Figure 2b). The paint layer is melted by the organic solvent in experiments for T=1 h (Figure 2a). Several bumps appear in experiments for T=56 h (Figure 2c), but they vanish at t=30 min, and a layer with a smooth surface remains. In experiments for T=64 h, the surface of the layer remains smooth and does not change with time (Figure 2d). The experiments for T=56 and 64 h both result in smooth surfaces, even after the application of the organic solvent, but the processes by which the smooth surfaces are produced are different. These results show that buckles emerge only for a limited range of the curing times *T*. This behavior is similar to the results obtained in previous experiments with double coatings [1,7].

#### 2.1.2. Characteristic Spatial Scales and Timescales for the Formation of Wrinkles

**Figure 3:**
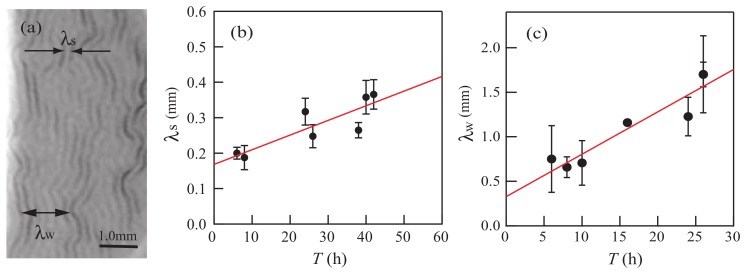
(**a**) Snapshot taken at t= 63 s in the experiment with elapsed time T=24 h. The short-scale wavelength $\lambda_{s}$ of the small wrinkles and the wavelength $\lambda_{w}$ of the larger-scale wrinkles are indicated. The solid line in the photo shows a 1.0-mm-scale bar. Panels (**b**,**c**) show the quantities $\lambda_{s}$ and $\lambda_{w}$ obtained from our experiments for several different values of *T*. The closed circles are the experimental data, and the solid lines in (**b**,**c**) are the fitted lines given by Equations (1) and (2), respectively.

Figure 3a shows the short-scale wavelength $\lambda_{s}$ and the wavelength $\lambda_{w}$ of larger-scale wrinkles obtained from experiments with T=24 h, which were mentioned in Section 2.1.1. Small structures with wavelengths $\lambda_{s}$ appear first, and wrinkles with wavelengths $\lambda_{w}$ appear subsequently. The quantity $\lambda_{w}$ is the maximum wavelength observed before the wrinkles coalesce. Figure 3b,c shows the values of $\lambda_{s}$ and $\lambda_{w}$, respectively, in experiments for several different values of *T*. We assume that they can be fitted by the linear functions

(1) $$\begin{array}{cc} \lambda_{s} & =c_{s1}T+c_{s2}, \end{array}$$

(2) $$\begin{array}{cc} \lambda_{w} & =c_{w1}T+c_{w2} \end{array}$$

with the fitting parameters $c_{s1}=0.41\times 10^{-2}$, $c_{s2}=0.17$, $c_{w1}=0.44\times 10^{-1}$, and $c_{w2}=0.37$. Using Equations (1) and (2), we can determine $\lambda_{s}$ and $\lambda_{w}$ for any value of *T*.

**Figure 4:**
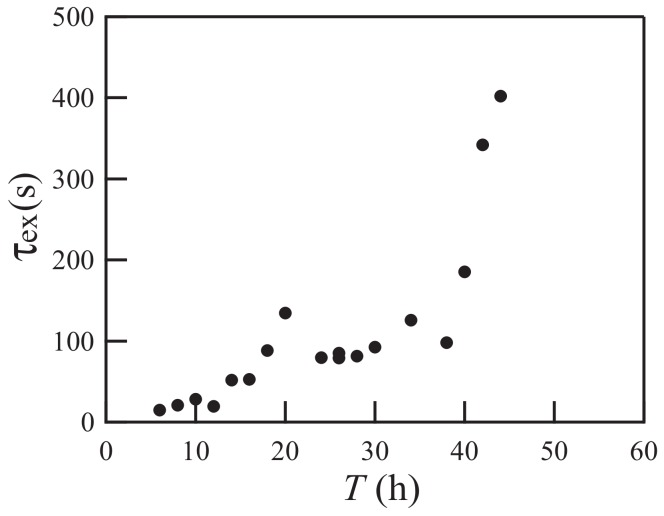
Relationship between *T* and $\tau_{\mathrm{ex}}$, where $\tau_{\mathrm{ex}}$ is the time elapsed between the application of the organic solvent and the appearance of bumps of 0.2 mm diameter.

Next, we investigate the characteristic timescale $\tau_{\mathrm{ex}}$, which turns out to be inversely related to the growth rate of the wrinkles. We define $\tau_{\mathrm{ex}}$ as the time elapsed between the application of the organic solvent and the appearance of bumps of 0.2 mm in diameter. As shown in Figure 4, $\tau_{\mathrm{ex}}$ increases with *T*. The timescale $\tau_{\mathrm{ex}}$ is larger than the times at which $\lambda_{s}$ and $\lambda_{w}$ are measured. After the initial growth of patterns with wavelengths $\lambda_{s}$ and $\lambda_{w}$, the coalescence of wrinkles is caused by nonlinear effects. Coalescence leads to a change in the characteristic length of pattern deformation. The time when such a change occurs is proportional to the timescale $\tau_{\mathrm{ex}}$ [8].

### 2.2. Model

**Figure 5:**
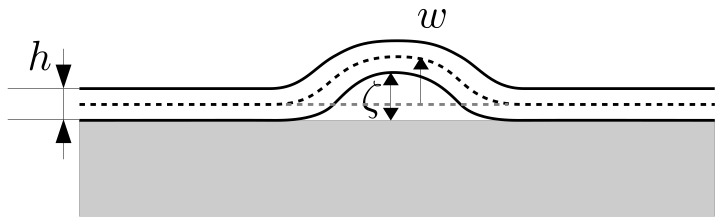
Schematic of a thin film on a substrate. The thickness of film is *h*. The mid-plane displacement and the distance between the substrate and film are denoted by *w* and ζ, respectively.

We next introduce a simple model for the buckling that is observed in our experiments. Consider the coating of paint to be an elastic thin film that is attached adhesively to a solid substrate (Figure 5). Because of the absorption of the organic solvent, the elastic film swells, producing compression stress. Suppose that the elastic film exists on a flat substrate, whose surface corresponds to the $x_{1}$-$x_{2}$ plane, and let the $x_{3}$ axis be normal to the surface of the substrate. The total energy $F_{\mathrm{tot}}$ of this system consists of the elastic strain energy in the film and the interfacial traction energy between the film and substrate:

(3) $$\begin{array}{c} F_{\mathrm{tot}}=\int \int \left(f_{\mathrm{film}}+f_{\mathrm{int}}\right)\;dx_{1}dx_{2}, \end{array}$$

where $f_{\mathrm{film}}$ and $f_{\mathrm{int}}$ are the energies per unit area of the film and the interface, respectively.

According to the Föppl–von Kármán plate theory, the elastic energy per unit area in a film of thickness *h* is given by [8,9,10,11,12]

(4) $$\begin{array}{cc} f_{\mathrm{film}} & =\int_{-h/2}^{h/2}\frac{1}{2}\sigma_{\alpha \beta }^{\mathrm{el}}\varepsilon_{\alpha \beta }^{\mathrm{el}}\;dx_{3}, \end{array}$$

(5) $$\begin{array}{cc} \sigma_{\alpha \beta }^{\mathrm{el}} & =\frac{2\mu }{1-\nu }\left[\left(1-\nu \right)\varepsilon_{\alpha \beta }^{\mathrm{el}}+\nu \varepsilon_{\gamma \gamma }^{\mathrm{el}}\delta_{\alpha \beta }\right], \end{array}$$

(6) $$\begin{array}{cc} \varepsilon_{\alpha \beta }^{\mathrm{el}} & =e_{\alpha \beta }-x_{3}\frac{\partial^{2}w}{\partial x_{\alpha }\partial x_{\beta }}. \end{array}$$

Greek subscripts refer to in-plane coordinates $x_{1}$ or $x_{2}$, and repeated Greek subscripts indicate summation over indices 1 and 2. The parameters μ and ν are the shear modulus and Poisson ratio of the film, respectively. Mid-plane displacements in the in-plane and $x_{3}$ directions are denoted by $u_{\alpha }$ and *w*, respectively. Supposing the film to be under equibiaxial stress, we take the initial in-plane strain to be $\varepsilon_{0}\delta_{\alpha \beta }$. Then,

(7) $$\begin{array}{c} e_{\alpha \beta }=\frac{1}{2}\left(\frac{\partial u_{\alpha }}{\partial x_{\beta }}+\frac{\partial u_{\beta }}{\partial x_{\alpha }}\right)+\frac{1}{2}\frac{\partial w}{\partial x_{\alpha }}\frac{\partial w}{\partial x_{\beta }}-\varepsilon_{0}\delta_{\alpha \beta }. \end{array}$$

To express the interfacial traction energy between the film and substrate, we use the cohesive zone model [13,14,15]. The interfacial energy per unit area is then

(8) $$\begin{array}{c} f_{\mathrm{int}}=\int_{0}^{\zeta }T_{n}\left(z\right)\;dz, \end{array}$$

where ζ is the distance between the substrate and film, and $T_{n}$ is the normal traction. When the film thickness is constant, ζ=w. We represent the normal traction as

(9) $$\begin{array}{c} T_{n}\left(\zeta \right)=\Gamma_{n}\zeta \exp\left(-\frac{\zeta }{\delta_{n}}\right), \end{array}$$

where $\Gamma_{n}\equiv \gamma_{n}/\delta_{n}^{2}$. The parameters $\gamma_{n}$ and $\delta_{n}$ are the normal interfacial toughness and the characteristic length of a normal displacement jump, respectively.

The total energy $F_{\mathrm{tot}}$ is thus expressed in terms of the displacements $u_{\alpha }$ and *w*. Equilibrium states must satisfy $\delta F_{\mathrm{tot}}/\delta w=0$ and $\delta F_{\mathrm{tot}}/\delta u_{\alpha }=0$. However, instead of solving $\delta F_{\mathrm{tot}}/\delta w=0$, we employ the time-dependent Ginzburg–Landau equation, which is often used in dynamical systems,

(10) $$\begin{array}{c} \frac{\partial w}{\partial t}=-\eta \frac{\delta F_{\mathrm{tot}}}{\delta w}, \end{array}$$

where η is a constant related to the characteristic relaxation time. Scaling all lengths by *h*, times by h/(μη), the nondimensional equation and $\gamma_{n}$ by hμ in Equation (10), we obtain

(11) $$\begin{array}{c} \frac{\partial w}{\partial t}=-\frac{1}{6(1-\nu )}\nabla^{2}\nabla^{2}w+\frac{\partial N_{\beta }}{\partial x_{\beta }}-T_{n}^{\prime }, \end{array}$$

where the variables are dimensionless, $T_{n}^{\prime }$ is the nondimensional form of Equation (9), and

(12) $$\begin{array}{cc} N_{\beta } & =\sigma_{\alpha \beta }\frac{\partial w}{\partial x_{\alpha }}, \end{array}$$

(13) $$\begin{array}{cc} \sigma_{\alpha \beta } & =\frac{2}{1-\nu }\left[\left(1-\nu \right)e_{\alpha \beta }+\nu e_{\gamma \gamma }\delta_{\alpha \beta }\right]. \end{array}$$

The in-plane displacements $u_{\alpha }$ included in $e_{\alpha \beta }$ are obtained from the equation $\delta F_{\mathrm{tot}}/\delta u_{\alpha }=0$.

### 2.3. Linear Stability Analysis

A linear stability analysis of Equation (11) provides some insight into the condition of buckling. Linearizing Equation (11) around w=0, and taking the Fourier transform of the linearized equation, we obtain

(14) $$\begin{array}{c} \frac{\partial \tilde{w}\left(k\right)}{\partial t}=g\left(k\right)\tilde{w}\left(k\right), \end{array}$$

where $\tilde{w}$ is the Fourier transform of *w*, and *k* is the wavenumber. The linear growth rate *g* is given by

(15) $$\begin{array}{c} g\left(k\right)=-\frac{1}{6(1-\nu )}{\left[k^{2}-6\left(1+\nu \right)\varepsilon_{0}\right]}^{2}+\frac{6{\left(1+\nu \right)}^{2}}{1-\nu }\varepsilon_{0}^{2}-\Gamma_{n}. \end{array}$$

Unstable modes, which cause deformations in the layer, appear when g(k)>0; in other words,

(16) $$\Gamma_{n}<\frac{6{\left(1+\nu \right)}^{2}}{1-\nu }\varepsilon_{0}^{2}.$$

This equation shows that wrinkles emerge above a certain threshold of stress. The existence of the threshold is consistent with the experimental results shown in Figure 2, which indicate that buckles emerge under an upper limit of *T*, since $\Gamma_{n}$ and $\varepsilon_{0}$ depend on *T*. Equation (15) shows that the wavenumber of the fastest-growing mode is

(17) $$\begin{array}{c} k_{f}=\sqrt{6\left(1+\nu \right)\varepsilon_{0}}. \end{array}$$

The growth rate of the fastest-growing mode is inversely proportional to the timescale,

(18) $$\begin{array}{c} \tau_{f}={\left[\frac{6{\left(1+\nu \right)}^{2}}{1-\nu }\varepsilon_{0}^{2}-\Gamma_{n}\right]}^{-1}. \end{array}$$

### 2.4. Numerical Simulations

**Figure 6:**
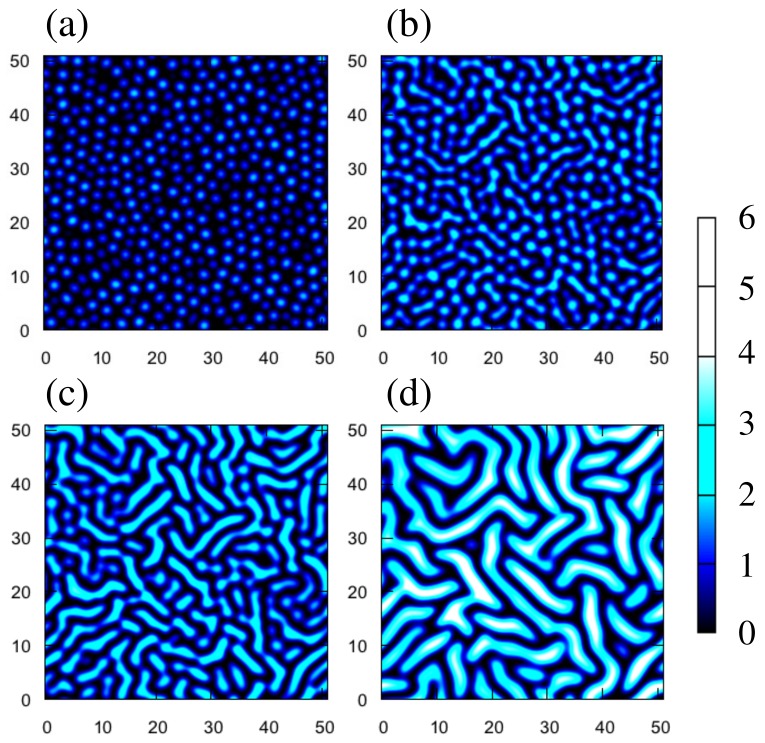
Snapshots of numerical simulations at (**a**) t=120, (**b**) 160, (**c**) 200, and (**d**) 400. The color scale illustrates the mid-plane displacement *w*. Panels (**a**,**b**) correspond to (**c**–**f**) of Figure 1, respectively. The length of a side of a snapshot corresponds to 6.8 mm when the film thickness is h=0.13 mm.

Numerical simulation is useful for demonstrating that our simple model does reproduce buckling of the film. Simulated patterns of the displacement *w* are shown in Figure 6. In the initial states, we take w=0 plus a small amount of noise, and we impose periodic boundary conditions on a 256×256 grid system. The length of a side corresponds to about 6.8 mm for h=0.13 mm. The values of the side length and *h* are close to experimental ones. In Figure 6, we take $\varepsilon_{0}=1.2$, $\Gamma_{n}=1.4$. The other parameters used in the following simulations are ν=0.3 and $\delta_{n}=0.5$.

Some characteristics of the snapshots in Figure 6 look similar to those of the experiments in Figure 1c–f. Small bumps appear at an early stage (Figure 6a). The amplitudes of the bumps grow with time, and some bumps coalesce with others (Figure 6b,c). However, the amplitudes continue to grow in the simulations (Figure 6d), which is significantly different from the experiments. This indicates that our model is not yet adequate to explain the nonlinear effects in the actual experiments.

**Figure 7:**
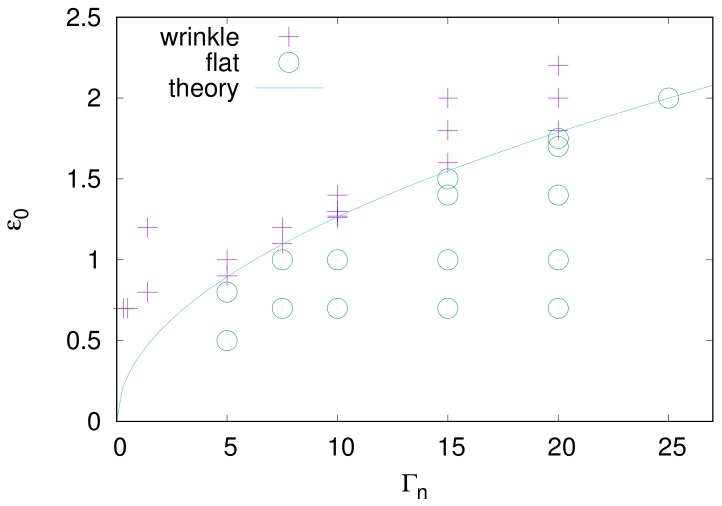
Phase diagram of wrinkle emergence. The horizontal and vertical axes are the parameter relating to the interfacial toughness and the initial in-plane strain, respectively. The solid curve corresponds to Equation (16), which is given by the linear stability analysis. Symbols are data of numerical simulations.

The phase diagram shown in Figure 7 illustrates the numerical verification of the wrinkle-emerging condition. The linear stability analysis suggests that wrinkles should appear above the solid curve, which is given by Equation (16). Numerical data shown as symbols demonstrate that the analysis is sufficiently valid.

**Figure 8:**
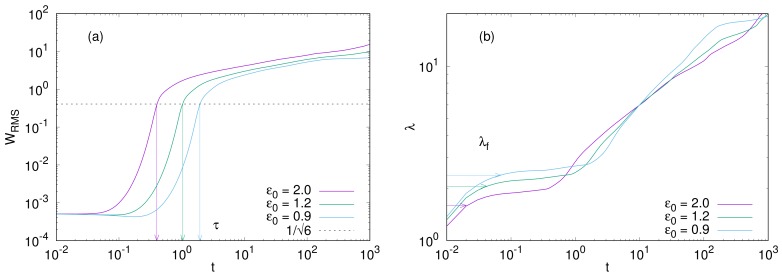
Time evolution of (**a**) the root mean square of *w* (related to the amplitude of wrinkles,**b**) the average wavelength of wrinkles. The arrows in (**a**) indicate the time when $W_{\mathrm{RMS}}\;=\;1/\sqrt{6}$. The arrows in (**b**) indicate the wavelength $\lambda_{f}=2\pi /k_{f}$ of the fastest mode, which is given by Equation (17).

Figure 8a shows the time evolution of the root-mean-square (RMS) of *w*,

(19) $$\begin{array}{c} W_{\mathrm{RMS}}=\sqrt{\frac{\int w{\left(k\right)}^{2}d^{2}r}{L^{2}}}, \end{array}$$

where *L* is the system size. The parameter values except for $\varepsilon_{0}$ are taken as the same as Figure 6. This is also interpreted as the time evolution of the average amplitude *A* of wrinkles, since $W_{\mathrm{RMS}}=\sqrt{1/2}A$, where we suppose a stripe form of $w=A\sin(k_{f}x_{1})$. The arrows in Figure 8a indicate the initial-growth time τ which is the transition time from the initial-growth regime to the coarsening one. Let us here estimate the amplitude at the initial-growth time. In the coarsening regime, we can assume that the interfacial energy becomes negligible and that the time evolution slows down significantly. Thus, setting $\partial w/\partial t=T_{n}^{\prime }=0$ in Equation (11), we have

(20) $$\begin{array}{c} \sigma_{11}=-\frac{k_{f}^{2}}{6(1-\nu )}. \end{array}$$

On the other hand, calculating the spacial average of $\sigma_{11}$ given by Equation (13) leads to

(21) $$\begin{array}{c} {\bar{\sigma }}_{11}=\frac{2}{1-\nu }\left[\frac{A^{2}}{4}k_{f}^{2}-\left(1+\nu \right)\varepsilon_{0}\right]=\frac{\left(3A^{2}-2\right)k_{f}^{2}}{6(1-\nu )}. \end{array}$$

Equating Equtaions (20) and (21), we have $A=\sqrt{1/3}$. The initial-growth time τ is determined as the time when $A=\sqrt{1/3}$, and thus, $W_{\mathrm{RMS}}=1/\sqrt{6}$.

The average wavelength of wrinkles is $\lambda =2\pi /\bar{k}$, where

(22) $$\begin{array}{c} \bar{k}=\sqrt{\frac{\int k^{2}{\left|\tilde{w}\left(k\right)\right|}^{2}d^{2}k}{\int |\tilde{w}{\left(k\right)|}^{2}d^{2}k}}. \end{array}$$

Here, $\tilde{w}$ is the Fourier transform of $w-\bar{w}$, where $\bar{w}$ is the spacial average of *w*. The time evolution of the average wavelength is shown in Figure 8b. In the initial-growth regime, the average wavelength is approximately equal to (or slightly larger than) that of the fastest-growing mode, which is indicated by the arrows in Figure 8b. The wavelength $\lambda_{f}$ of the fastest-growing mode is estimated from the linear stability analysis and evaluated as $\lambda_{f}=2\pi /k_{f}$, where $k_{f}$ is given by Equation (17).

### 2.5. Correspondence between Experimental and Numerical Results

We here rewrite the time scale $\tau_{f}$ in other forms to examine experimental and numerical results by means of the linear stability analysis. Suppose that the growth rate of a certain unstable mode $k_{1}$ is $g(k_{1})=C$, where *C* is a positive constant. Using Equations (15), (17) and (18), we have

(23) $$\begin{array}{c} {k_{f}}^{2}-k_{1}^{2}=\sqrt{6\left(1-\nu \right)\left(\tau_{f}^{-1}-C\right)}, \end{array}$$

where $k_{1}<k_{f}$. Equation (23) leads to

(24) $$\begin{array}{c} \tau_{f}=\frac{6(1-\nu )}{{\left(k_{f}^{2}-k_{1}^{2}\right)}^{2}+C^{\prime }}\propto {\left[{\left(\frac{1}{\lambda_{f}^{2}}-\frac{1}{\lambda_{1}^{2}}\right)}^{2}+C^{\prime }\right]}^{-1}, \end{array}$$

where $k_{f}=2\pi /\lambda_{f}$, $k_{1}=2\pi /\lambda_{1}$, and $C^{\prime }$ is a constant. For the minimum wavenumber $k_{\min}$ with a non-negative growing rate, $g(k_{\min})=0$, and thus, $C=C^{\prime }=0$. Then, Equtaions (23) and (24) turn to be

(25) $$\begin{array}{c} \tau_{f}=\frac{6(1-\nu )}{{\left(k_{f}^{2}-k_{\min}^{2}\right)}^{2}}\propto {\left(k_{f}^{2}-k_{\min}^{2}\right)}^{-2}, \end{array}$$

where

(26) $$\begin{array}{c} k_{f}^{2}-k_{\min}^{2}=\sqrt{6\left(1-\nu \right)\left(\frac{6{\left(1+\nu \right)}^{2}}{1-\nu }\varepsilon_{0}^{2}-\Gamma_{n}\right)}. \end{array}$$

Equations (24) and (25) are useful to examine experimental and numerical results, respectively.

We first examine experimental results, using Equation (24). We assume that $\lambda_{f}$ and $\lambda_{1}$ in Equation (24) correspond to $\lambda_{s}$ and $\lambda_{w}$ in Figure 3, respectively. This assumption implies that structures with wavelengths $\lambda_{s}$ and $\lambda_{w}$ appear in the linear-instability region and that $\lambda_{s}$ and $\lambda_{w}$ correspond to unstable modes of pattern formation. We also assume that $\tau_{\mathrm{ex}}\propto \tau_{f}$ [8].

**Figure 9:**
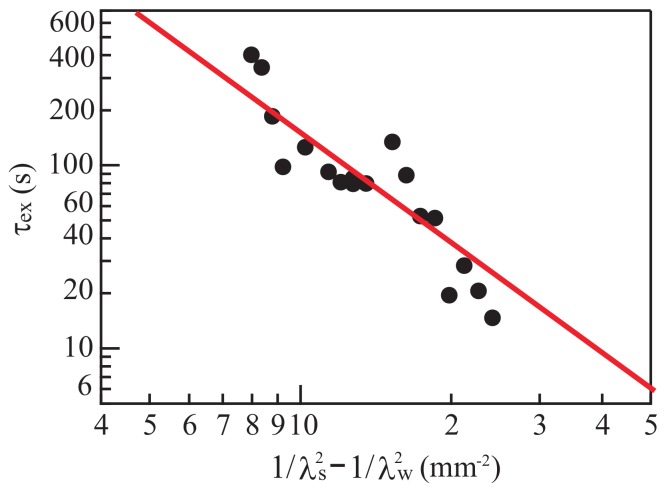
The relationship between $\tau_{\mathrm{ex}}$ and $\left(1/\lambda_{s}^{2}\right)-\left(1/\lambda_{w}^{2}\right)$, where $\tau_{\mathrm{ex}}$ is the time between the application of the organic solvent and the appearance of bumps of 0.2 mm diameter. The quantities $\lambda_{s}$ and $\lambda_{w}$ are the wavelengths of the small wrinkles that first appear and the maximum wavelength observed before the wrinkles coalesce, respectively. The closed circles are the experimental results, and the solid line is the fit from Equation (24).

The closed circles in Figure 9 show experimental data about the relation between $\tau_{\mathrm{ex}}$ and $\left(1/\lambda_{s}^{2}\right)-\left(1/\lambda_{w}^{2}\right)$. Values of $\lambda_{s}$ and $\lambda_{w}$ are obtained using Equations (1) and (2). The experimental data agree reasonably closely with the line given by Equation (24), which is shown as a solid line. The exponent of the fitted line is −2, and $C^{\prime }$ of Equation (24) nearly vanishes for the fitted line.

**Figure 10:**
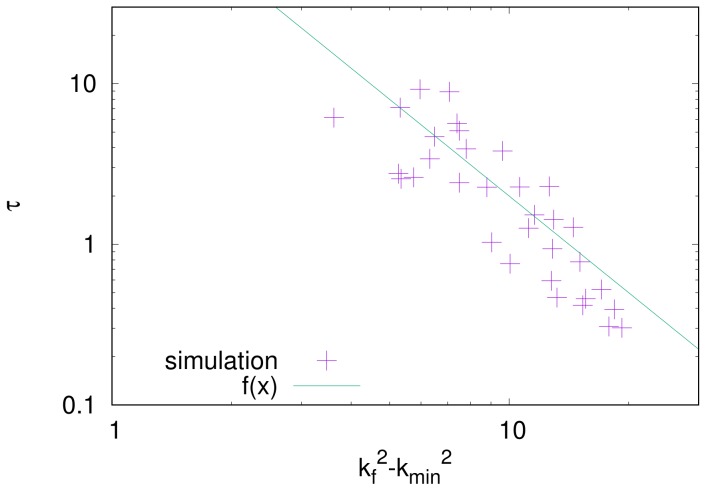
Initial-growth time τ as a function of $k_{f}^{2}-k_{\min}^{2}$, where $k_{f}$ and $k_{\min}$ denote the wavenumber of the fastest-growing mode and the minimum wavenumber with a non-negative growth rate, respectively. Symbols are the numerical results, and the solid line is the fit from Equation (25).

Figure 10 shows the numerical counterparts. The initial-growth time τ is plotted as a function of $k_{f}^{2}-k_{\min}^{2}$. The initial-growth time is defined as the time when $W_{\mathrm{RMS}}$ reaches $1/\sqrt{2}$ (See Figure 8), and its value is obtained from numerical simulations with different combinations of $\varepsilon_{0}$ and $\Gamma_{n}$. The parameters used in the simulation gives $k_{f}^{2}-k_{\min}^{2}$ through Equation (26). Assuming that the initial-growth time τ is proportional to $\tau_{f}$ of Equation (25), we fit the line given by Equation (25) to the numerical data. The fitted line reasonably agrees with the numerical data.

## 3. Conclusions

The objective of this paper has been to understand the emergence of wrinkles at the surface of a coating following the application of an organic solvent. The instability at the surface of the layer leads to the emergence of wrinkles. We investigated the characteristic lengths of the wrinkles and the characteristic timescale for wrinkle emergence in experiments and numerical simulations. The linear stability analysis of our simple model supports the experimental and numerical results. Although the simple model suitably explains the emergence of wrinkles, we will need a more realistic model to investigate the coarsening of wrinkles and time evolution of wrinkle patterns. For example, the film thickness, the strain induced by volume expansion and the interfacial traction vary with time in experiments as the solvent evaporates. Those effects should be included in the model to investigate wrinkle patterns beyond the linear-stability regime.

Our results indicate that the initial strain $\varepsilon_{0}$ and the interfacial toughness $\Gamma_{n}$ depend on the curing time *T*. Although the dependencies have not been specified yet, our results will be useful especially in engineering. For example, even if *T* is unknown, we can estimate $\varepsilon_{0}$ and $\Gamma_{n}$ from Equtaions (17) and (18) by measuring the wavelength of wrinkles and $\tau_{\mathrm{ex}}$. Those parameters are essential for the control of wrinkle formation.

We conclude that (1) buckling due to volume expansion of the layer and (2) delamination of the layer from the substrate are both important for the formation of wrinkles. This conclusion is supported by the linear stability analysis which states that the emergence of wrinkles depends on both the initial strain caused by volume expansion and the normal traction. Experimental results as well as numerical ones show reasonably good agreement with the linear stability analysis.

## 4. Materials and Methods

### 4.1. Experimental Method

**Figure 11:**
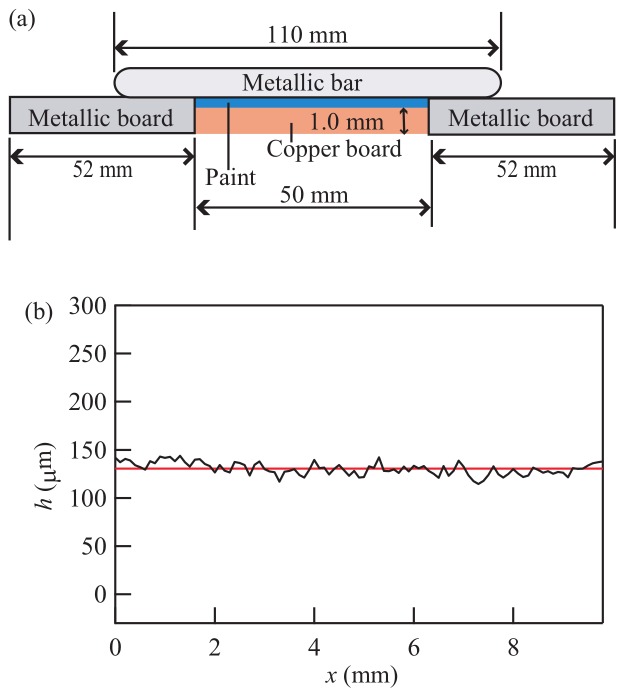
(**a**) Schematic drawing of the experimental setup for the application of a paint layer. Only the coated copper board is kept in a constant-temperature oven for several hours after the first coating. (**b**) The surface height *h* measured after painting.

We used a copper board of 5.0 cm × 5.0 cm square and 1.0 mm thick as the substrate for painting. To control the thickness of the paint layer, we placed two metallic boards facing each other on opposite sides of the copper board, as shown in Figure 11a. The metallic boards are of equal thickness and are slightly thicker than the copper board. We applied a phthalic resin paint (Rubicon1000, No. 837, ISHIKAWA PAINT) to the copper board using a syringe (SS-20ESZ, TERMO), and we spread the paint across the copper board using a metallic bar, producing a layer of relatively uniform thickness. We measured the thickness *h* of the paint layer using a laser displacement meter (LT9010M, KEYENCE). As shown in Figure 11b, we found that the layer had a nearly uniform thickness with an average value h=130±6μm. The coated board was then placed in a constant-temperature oven (NEXAS OFX-70, ASONE) at 30 °C for a time *T*. After the time *T*, we applied a 0.02 cm^3^ drop of xylene, which is the organic solvent in the paint, on the coated layer. The surface of the layer was photographed with a digital camera (Canon EOS Kiss X4, EF-S 18-55IS) 10 min after the xylene application. It was easy to observe the deformations of the paint layer, since xylene is clear and colorless.

### 4.2. Numerical Procedure

We employed a spectral method for numerical simulations. The Fourier transform of Equation (11) is

(27) $$\begin{array}{c} \frac{\partial \tilde{w}}{\partial t}=-Dk^{4}\tilde{w}-ik_{\beta }{\tilde{N}}_{\beta }-{\tilde{T}}_{n}, \end{array}$$

where D=1/[6(1−ν)] and ${\tilde{N}}_{\beta }$ and ${\tilde{T}}_{n}$ are the Fourier transforms of Equation (12) and of the normal traction $T_{n}^{\prime }$, respectively.

The nonlinear term $N_{\beta }$ includes derivatives of $u_{\alpha }$. By using the condition $\delta F_{\mathrm{tot}}/\delta u_{\alpha }=0$, the Fourier transform of $u_{\alpha }$ can be written as

(28) $$\begin{array}{c} {\tilde{u}}_{\alpha }={\tilde{G}}_{\alpha \beta }{\tilde{\rho }}_{\beta }, \end{array}$$

where

(29) $$\begin{array}{cc} {\tilde{G}}_{\alpha \beta } & =\frac{1}{1-\nu }\left(\frac{\delta_{\alpha \beta }}{k^{2}}-\frac{1+\nu }{2}\frac{k_{\alpha }k_{\beta }}{k^{4}}\right), \end{array}$$

(30) $$\begin{array}{cc} {\tilde{\rho }}_{\alpha } & =\;\int \int \;\left[\left(1+\nu \right)\frac{\partial w}{\partial x_{\gamma }}\frac{\partial^{2}w}{\partial x_{\alpha }\partial x_{\gamma }}+\left(1-\nu \right)\frac{\partial w}{\partial x_{\alpha }}\nabla^{2}w\right]e^{ik\cdot r}dx_{1}dx_{2}. \end{array}$$

Using Equtaions (28)–(30), we can rewrite Equation (7) in the following form [11,12],

(31) $$\begin{array}{c} e_{\alpha \beta }=\frac{1}{2}\int_{k\neq 0}\left[-i\left(k_{\beta }{\tilde{G}}_{\alpha \gamma }+k_{\alpha }{\tilde{G}}_{\beta \gamma }\right){\tilde{\rho }}_{\gamma }\right]\frac{e^{-ik\cdot r}}{{\left(2\pi \right)}^{2}}d^{2}k+\frac{1}{2}\frac{\partial w}{\partial x_{\alpha }}\frac{\partial w}{\partial x_{\beta }}-\varepsilon_{0}\delta_{\alpha \beta }. \end{array}$$

In numerical simulations, we used the modified normal traction,

(32) $$\begin{array}{c} T_{n}\left(\zeta \right)=\left\{\begin{array}{cc} \Gamma_{n}\zeta \exp\left(-\zeta /\delta_{n}\right) & \zeta \geq 0,\\ \Gamma_{n}^{\prime }\zeta \exp\left(-\zeta /\delta_{n}\right) & \zeta <0, \end{array}\right. \end{array}$$

where $\Gamma_{n}^{\prime }$ is a parameter that is sufficiently larger than $\Gamma_{n}$. In the simulations, we set $\Gamma_{n}^{\prime }=100\;\Gamma_{n}$. Although Equation (9) is convenient for linear stability analysis, it is inconvenient for numerical simulations; if Equation (9) was used as the normal traction, areas with w<0 would appear. Since the substrate is solid, negative values of *w* are not allowed in realistic situations. The modified traction given by Equation (32) enables the calculations to avoid such unrealistic solutions.

For the time evolution, we employed a semi-implicit algorithm: we used first-order backward and forward finite-difference schemes for the linear and nonlinear parts of Equation (27), respectively. The (n+1)-th step in the calculation of $\tilde{w}$ is given by

(33) $$\begin{array}{c} {\tilde{w}}^{\left(n+1\right)}=\frac{{\tilde{w}}^{\left(n\right)}-\left(ik_{\beta }{\tilde{N}}_{\beta }^{\left(n\right)}+{\tilde{T}}_{n}^{\left(n\right)}\right)\Delta t}{1+Dk^{4}\Delta t}, \end{array}$$

where Δt is the time increment.
